# Supplementary material for: Content-rich biological network constructed by mining PubMed abstracts
Source: BMC Bioinformatics. 2004 Oct 8;5:147. doi: 10.1186/1471-2105-5-147 (PMC528731; doi:10.1186/1471-2105-5-147)
Supplement: Additional File 2 — The original results of the above study (non-essential files are deleted to keep the file size under the limit set by BMC bioinformatics). [file 1471-2105-5-147-S2.bz2 › chilibotAdditionalFile2/dip05/710ID11274135E18047/html/RELB_RELE.html]

 


 **RELB** and **RELE** 
  
Found 8 abstracts in PubMed, retrieved 05.  
 

 What does Google say? 
 PDF only 
| .edu only 

---

**Interactive relationship** (e.g. stimulation, inhibition, etc)

**Neutral relationship**- **RelB**  counteracts the toxic effect of  **RelE** .  Ref: 11717402 Proc Natl Acad Sci U S A, 2001
- Activation of  **RelE**  depends on Lon mediated degradation of  **RelB** , the antagonist that neutralizes  **RelE** .  Ref: 12787364 Mol Microbiol, 2003
- Later induction of transcription of the antitoxin genes  **relB**  or chpAI fully reversed the static condition induced by  **RelE**  and ChpAK respectively.  Ref: 12123459 Mol Microbiol, 2002
- After neutralization of  **RelE**  by  **RelB** , rapid recovery of translation required tmRNA, indicating that tmRNA alleviated  **RelE**  toxicity by rescuing ribosomes stalled on damaged mRNAs.  Ref: 12787364 Mol Microbiol, 2003
- **RelB**  can reverse inhibition of protein synthesis by  **RelE**  in vivo.  Ref: 12526800 Cell, 2003

**Non-interactive relationship** (e.g. studied together, co-existance, homology, etc.)

- It is ubiquitous in prokaryotes as part of the  **RelE**   **RelB**  toxin antitoxin system, and may be activated by nutritional stress.  Ref: 12023043 FEBS Lett, 2002
- The Escherichia coli relBE operon encodes a toxin antitoxin pair,  **RelE**   **RelB** .  Ref: 12526800 Cell, 2003
- The second rel locus of Escherichia coli is called relBE and encodes  **RelE**  cytotoxin and  **RelB**  antitoxin.  Ref: 11717402 Proc Natl Acad Sci U S A, 2001
- Replacement of the relBE promoter with a LacI regulated promoter indicated that strong and ongoing transcription of relBE is required to maintain a proper  **RelB**   **RelE**  ratio during starvation.  Ref: 11717402 Proc Natl Acad Sci U S A, 2001
